# Towards deep-learning (DL) based fully automated target delineation for rectal cancer neoadjuvant radiotherapy using a divide-and-conquer strategy: a study with multicenter blind and randomized validation

**DOI:** 10.1186/s13014-023-02350-0

**Published:** 2023-10-06

**Authors:** Jianhao Geng, Xianggao Zhu, Zhiyan Liu, Qi Chen, Lu Bai, Shaobin Wang, Yongheng Li, Hao Wu, Haizhen Yue, Yi Du

**Affiliations:** 1 Key laboratory of Carcinogenesis and Translational Research (Ministry of Education/Beijing), Department of Radiation Oncology, Peking University Cancer Hospital & Institute, Beijing, 100142 China; 2 Research and Development Department, MedMind Technology Co., Ltd, Beijing, 100083 China; 3 Institute of Medical Technology, Peking University Health Science Center, Beijing, 100191 China

**Keywords:** Rectal cancer radiotherapy, Deep learning, CTV, GTV, Segmentation

## Abstract

**Purpose:**

Manual clinical target volume (CTV) and gross tumor volume (GTV) delineation for rectal cancer neoadjuvant radiotherapy is pivotal but labor-intensive. This study aims to propose a deep learning (DL)-based workflow towards fully automated clinical target volume (CTV) and gross tumor volume (GTV) delineation for rectal cancer neoadjuvant radiotherapy.

**Materials & methods:**

We retrospectively included 141 patients with Stage II-III mid-low rectal cancer and randomly grouped them into training (n = 121) and testing (n = 20) cohorts. We adopted a divide-and-conquer strategy to address CTV and GTV segmentation using two separate DL models with DpuUnet as backend-one model for CTV segmentation in the CT domain, and the other for GTV in the MRI domain. The workflow was validated using a three-level multicenter-involved blind and randomized evaluation scheme. Dice similarity coefficient (DSC) and 95th percentile Hausdorff distance (95HD) metrics were calculated in Level 1, four-grade expert scoring was performed in Level 2, and head-to-head Turing test in Level 3.

**Results:**

For the DL-based CTV contours over the testing cohort, the DSC and 95HD (mean ± SD) were 0.85 ± 0.06 and 7.75 ± 6.42 mm respectively, and 96.4% cases achieved clinical viable scores (≥ 2). The positive rate in the Turing test was 52.3%. For GTV, the DSC and 95HD were 0.87 ± 0.07 and 4.07 ± 1.67 mm respectively, and 100% of the DL-based contours achieved clinical viable scores (≥ 2). The positive rate in the Turing test was 52.0%.

**Conclusion:**

The proposed DL-based workflow exhibited promising accuracy and excellent clinical viability towards automated CTV and GTV delineation for rectal cancer neoadjuvant radiotherapy.

## Introduction

Colorectal cancer is one of the top malignancies in terms of incidence and mortality not only in China [1] but also worldwide [2]. The proportion of rectal cancer in colorectal cancer is nearly 2/3, among which mid-low rectal cancer accounts for approximately 70% [3]. Unfortunately, most patients have already progressed into the advanced stages at the time of their initial diagnosis. A neoadjuvant chemoradiation regimen followed by surgery is the current standard of care for locally advanced mid-low rectal cancers [4–6].

Gross tumor volume (GTV) and clinical target volume (CTV) delineation by oncologists are critical steps in radiotherapy (RT) treatment planning. Accurate target delineation is vitally important in ensuring the delivery of a safe and effective radiation dose to the tumor while minimizing damage to surrounding healthy tissues. According to guidelines of rectal cancer RT [7–10], the GTV includes the visible and palpable tumor in the rectum as well as any metastatic lymph nodes, and the CTV includes the above GTV region plus any areas at risk for microscopic disease spread, such as the internal iliac, presacral, and perirectal nodal regions. Since the tissue contrast between computed tomography (CT) and magnetic resonance imaging (MRI) are substantially different, patients with locally advanced rectal cancer are routinely prescribed to take both CT and MRI simulations to facilitate accurate target delineation. Specifically, CT images are primarily used for CTV delineation, while GTV delineation is heavily reliant on MRI images for clear visualization of the primary site. Although dual imaging modalities are employed, the process of GTV and CTV delineation remains labor-intensive, with the slice-by-slice procedure often taking more than an hour for radiation oncologists to complete.

To achieve accurate and efficient target delineation for radiotherapy, growing efforts have been directed to automated segmentation methods, particularly with deep learning (DL) as backend. However, the work pertinent to rectal cancer target segmentation is rare. Men et al. [11, 12] designed a deep dilated CNN (DDCNN)-based model for CTV segmentation, which outperformed the classic Unet with 3.8% increase in Dice similarity coefficient (DSC). Larsson et al. [13] developed a three-dimensional Vnet model, exhibiting improved DSC values over both Unet and DDCNN. Also, Wu et al. [14] derived a network model from the classic Unet by adding more skip connections, the performance of which was validated by expert evaluation. Besides, Song et al. [15] used the DeepLabv3 + network for postoperative rectal cancer CTV segmentation. As for GTV, to date only Wang et al. [16] proposed to use Unet for GTV auto-segmentation for rectal cancer neoadjuvant radiotherapy. In addition, there are some attempts to auto-segment GTV for esophageal cancer [17, 18].

Moreover, to the best of our knowledge, we did not find any relevant work towards automated segmentation of rectal cancer CTV and GTV for neoadjuvant radiotherapy in the same process. This can be partly attributed to the aforementioned clinical routine practice that CTV and GTV are delineated using information from different imaging modalities. Despite this, the routine practice indicates the feasibility to fully automate the target delineation procedure by means of divide-and-conquer.

To this end, this is the first study that has developed a DL-based workflow towards fully automated CTV and GTV delineation for rectal cancer neoadjuvant radiotherapy. The workflow was based on the human reasoning process and the target segmentation task was performed in a divide-and-conquer strategy, i.e., CTV in CT domain and GTV in MRI domain respectively. To validate the workflow in more aspects than comparison with ground truth, we employed a multicenter-involved blind and randomized evaluation scheme.

## Materials & methods

### Data collection and preparation

As a plot study approved by the institutional review board a cohort of 141 patients treated at our institute (Peking University Cancer Hospital) between March 2020 and May 2022 were retrospectively included in this study. The patients were diagnosed with Stage II to III rectal cancer, and received neoadjuvant chemoradiotherapy, which is the standard treatment for locally advanced rectal cancer. The cohort were categorized into training group (121/141) and testing group (20/141) by random sampling (Fisher-yates shuffle). Over the training group, 69 were female and 52 were male, and the ages ranged from 33 to 74 with the median value of 61. Over the testing group, 13 were female and 7 were male, and the ages ranged from 39 to 72 with the median value of 63.

All the patients were immobilized by pelvis thermoplastic in the supine posture and received CT and MRI simulations respectively. The CT images were acquired on a big-bore RT-specific CT scanner (Somation Sensation Open, Siemens Healthineers, Germany) with 5-mm slice thickness, and MRI T2 and T1 images on a 3.0-T MR-Sim scanner (MAGNETOM Skyra, Siemens Healthineers, Germany) with 5-mm slice thickness as well. The CT and MRI images were imported into the Eclipse Treatment Planning System (Varian Medical System Inc., USA) for target and organs-at-risk (OAR) delineation. Due to the different imaging contrast properties between CT and MRI, CTV structures were contoured in CT images, and GTV structures in MRI T2 images. Note that the CTV and GTV definitions in this study were consistent with the NCCN and ESMO guideline. The CTV and GTV contours of all the patients were reviewed by two senior physicians, and therefore were used as ground truth (GT) herein.

### DL model for CTV and GTV segmentation

The kernel DL network herein were DpnUnet, a highly capable network that demonstrates impressive performance in segmentation tasks with fuzzy boundaries, and validated in cervical cancer for CTV and OAR segmentation [19]. It is important to note, since CTV and GTV structures were contoured in two disparate image domains, CT and MRI, we proposed to use a divide-and-conquer strategy: two DL models were built respectively to take in CT images for CTV segmentation and MRI images for GTV segmentation respectively and specifically. Despite the identical network architecture, the input, network parameters (weights) and output were completely different.

#### DpnUnet architecture

**Fig. 1:**
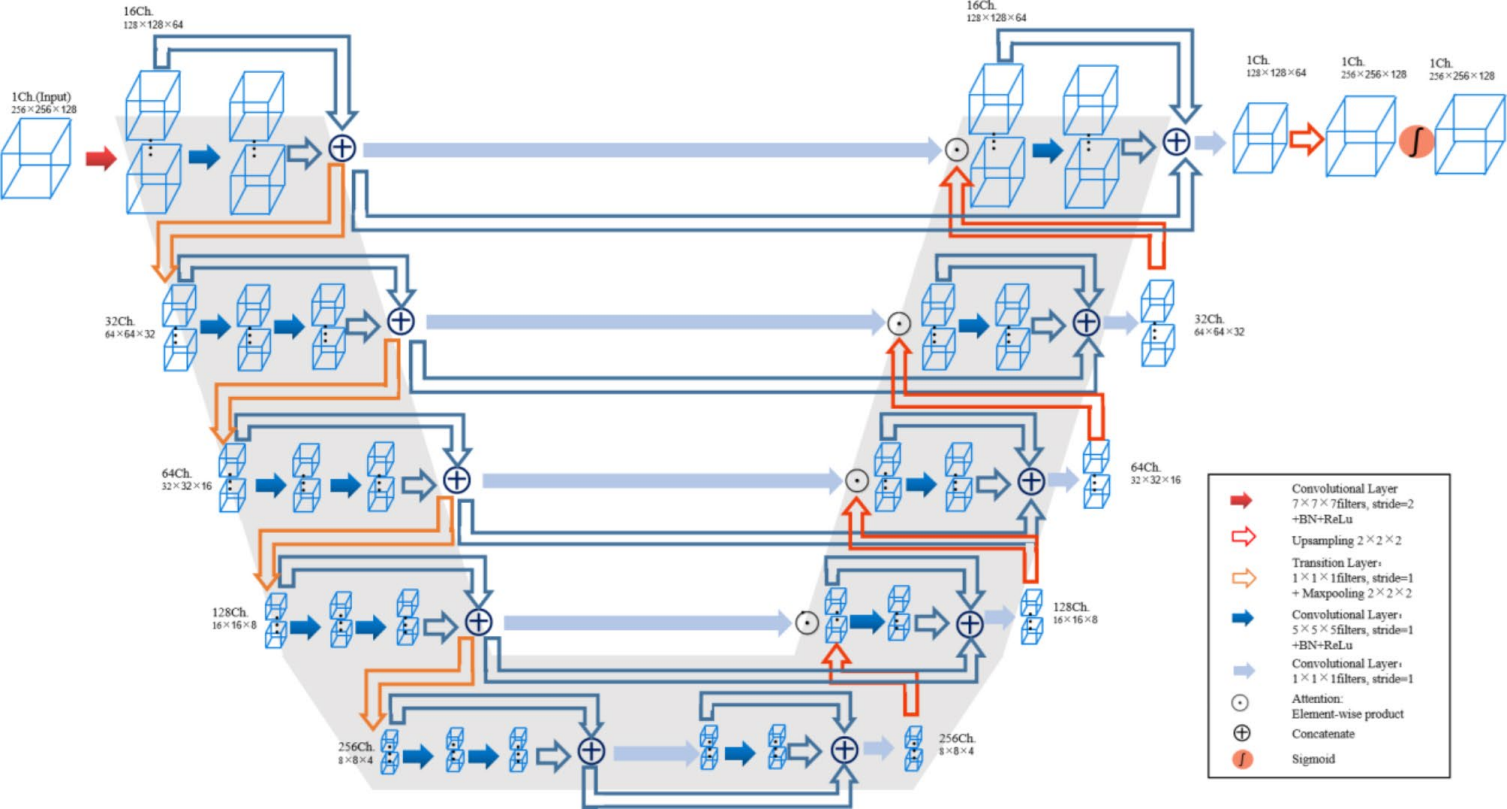
Schematic of the kernel DpnUnet network architecture

The DpnUnet was a Unet variant characterized by the typical U-shape encoder-decoder design and locally integrated with dual-path-network (DPN) modules. The overall architecture of the DpnUnet network was illustrated in Fig. 1. Briefly, the original U-net encoder part was replaced with the DPN92 model, and the decoder part embedded the micro-blocks in the DPN92 network to achieve paralleled performance in abstract feature recovery. The input layer took in 3 adjacent slices (empirical value) to incorporate 3D anatomy context, and the network delivered the predicted regions-of-interest (ROIs) in the middle slice. Generally, the DpnUnet network was an end-to-end segmentation framework that could achieve pixel-wise labeling in both CT and MRI images. Once the two models were trained, regions of CTV and GTV were auto-segmented slice by slice.

#### Model training

The training processes of CTV and GTV DpnUnet networks were the same but independently with different training data. There were 121 patient cases in the training group. The DpnUnet networks were trained with 11-fold (10:1) cross-validation, 110 cases for training and the other 11 cases for validation. Generic data augmentation techniques including flip, and translation, rotation were used. The networks were implemented by PyTorch 1.12.0 and Python 3.6, and trained on a NVIDIA P100 GPU (16 GB memory). Both of the CTV and GTV kernel networks were initialized with a pre-trained network that had been trained for OAR segmentation in cervical cancer CT images [20]. The optimizer was Adam. The learning rate was initialized as 0.0001 and decayed by an exponential function with gamma 0.9 for every epoch. The total epoch number was 100 with the batch size as 4, and the model with the lowest validation loss was selected as the output for further testing. The optimizer, learning rate and batch size were also the same for both CTV and GTV model training.

### Performance evaluation

**Fig. 2:**
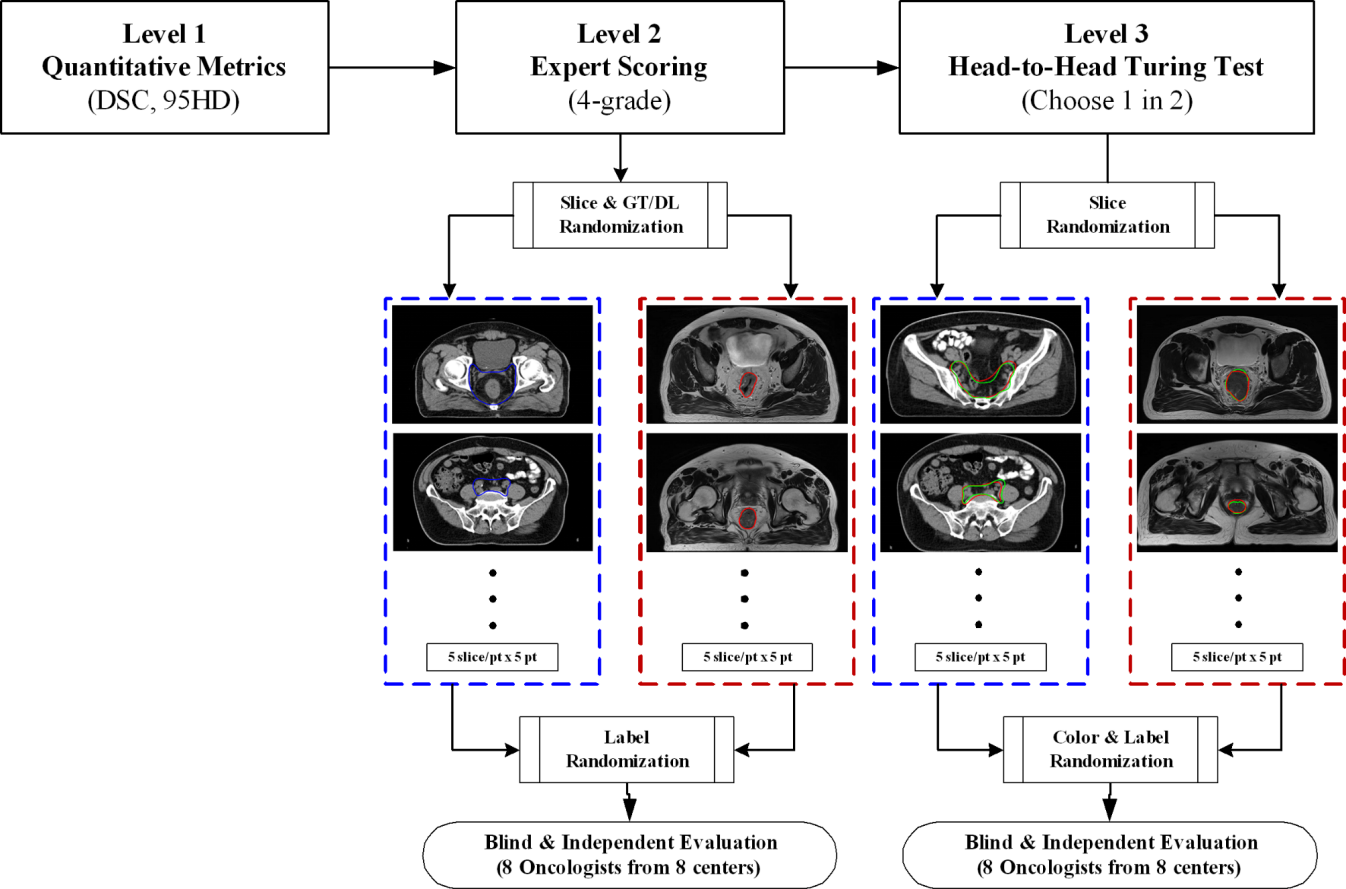
3-Level evaluation design for DL-based CTV and GTV auto-segmentation

There were 20 patient cases in the testing group. We adopted the three-level evaluation design proposed by [14] to assess both the CTV and GTV DL model performance in more aspects than one. The evaluation procedure was depicted in Fig. 2. The Level-1 evaluation focused on objective metrics, and the Level-2 and Level-3 focused on oncologists’ subjective assessment of clinical viability. Moreover, to enhance the generalizability of subjective evaluation of the proposed method, we invited 8 senior radiation oncologists from 8 different cancer centers to score contours blindly and independently.

#### Level 1: quantitative metrics based objective evaluation

The Dice similarity coefficient (DSC) and 95th percentile Hausdorff distance (95HD) [21] were used in Level-1 to quantify the contouring accuracy. The DSC index defined in Eq. (1) was to measure the relative volumetric overlap between two contours and the value equals 1 when two contours are completely the same.

1 $$\mathbf{DSC}\mathbf{(P,G)}\mathbf=\frac{\mathbf{2}\left|\mathbf{P}\cap\mathbf{G}\right|}{\left|\mathbf{P}\right|+\left|\mathbf{G}\right|}$$

Where *P* and *G* represented the predicted and ground contours respectively, and |P∩G| represented the volume that *P* and *G* intersected.

The 95HD index defined in Eqs. (2–3) was to reflect the overlapping between two contours by mismatching distance, and higher distance value indicates larger contour difference.

2 $$\mathbf{95HD}\mathbf{(P},\mathbf{G)}\mathbf=\mathbf{percentile}\mathbf{(h}\mathbf{(P,G)}\cup\mathbf{h}\mathbf{(G,P),}\ \mathbf{95th})$$

3 $$\mathbf{h}(\mathbf{P},\mathbf{G})=\mathbf{max}(\mathbf{min}\mathbf{||p-g||}),\mathbf{p?P,g?G}$$

Where ||.|| is the Euclidean norm of the points of *p* and *g*.

The DSC and 95HD values were calculated in each testing case, as well as the mean and standard deviation (SD) over the entire testing group.

#### Level 2: blind & randomization expert scoring

Ten out of the 20 testing patients were randomly selected by Fisher-Yates shuffle for Level-2 evaluation. For CTV evaluation, five patients were randomly selected, and for each patient five CT slices were selected to display GT contours (CTV-GT: 5 × 5 = 25), and it went likewise with the rest 5 patients to generate CT slices with DL contours (CTV-DL: 5 × 5 = 25 slices in one folder). Similarly, five MRI slices for five randomly selected patients were randomly extracted to display GT GTV contours (GTV-GT: 5 × 5 = 25) and five MRI slices for the rest 5 patients to display DL GTV contours (GTV-DL: 5 × 5 = 25 slices in another folder). The DICOM-RT slices were exported as non-compressed TIFF images. In total, two folders of 50 images for CTV (GT = 25, DL = 25) and GTV (GT = 25, DL = 25) evaluation were prepared. The images in each folder were reshuffled (in Python) and anonymized by ordering numbers each time before we sent them to an external expert for independent scoring.

The rubric for scoring was in grade (Table 1): 3 for Accept, 2 for Minor Revision, 1 for Major Revision and 0 for Reject. The scores ≥ 2 were defined to be viable for clinical application. In addition, the scores in the GT and DL groups were statistically compared by Mann-Whitney U-test (significant level: p < 0.05).

**Table 1:** Rubric for expert scoring

Score	Grade	Criteria
3	Accept	The segmentation is acceptable for clinical treatment.
2	Minor Revision	A few minor edits of the segmentation are recommended, while the clinical impact may be not significant.
1	Major Revision	A few major edits of the segmentation are mandatory.
0	Reject	The segmentation is rejected and a redrawing is required.

#### Level 3: blind & randomization based head-to-head turing test

The rest ten testing patients were used for Level-3 evaluation. For each testing patient, five CT slices were randomly selected to display both CTV-GT and CTV-DL contours simultaneously (CTV = 10 × 5), and likewise five MRI slices to display both GTV-GT and GTV-DL contours (GTV = 10 × 5). In total, two folders of 50 slices for CTV and GTV evaluation were prepared. The DL and GT contour colors (red/green) in each image was randomized (Fisher-yates shuffle), and the images in each folder were reshuffled by *random.shuffle()* in Python and anonymized by ordering numbers, each time before the dataset was distributed along with the Level-2 dataset.

For each testing image, external experts were required to choose the optimal contour (positive) for clinical application. The positive rates of CTV-DL and GTV-DL contours were calculated, and the threshold for passing the Turing test was 30%, an empirical value [22].

## Results

### Level 1: DSC and 95HD

**Fig. 3:**
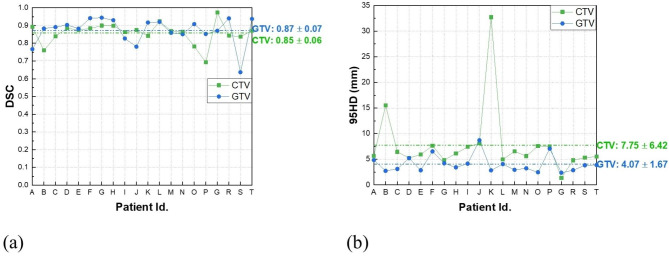
DSC and 95HD distribution over the testing cohort (20 patients)

**Fig. 4:**
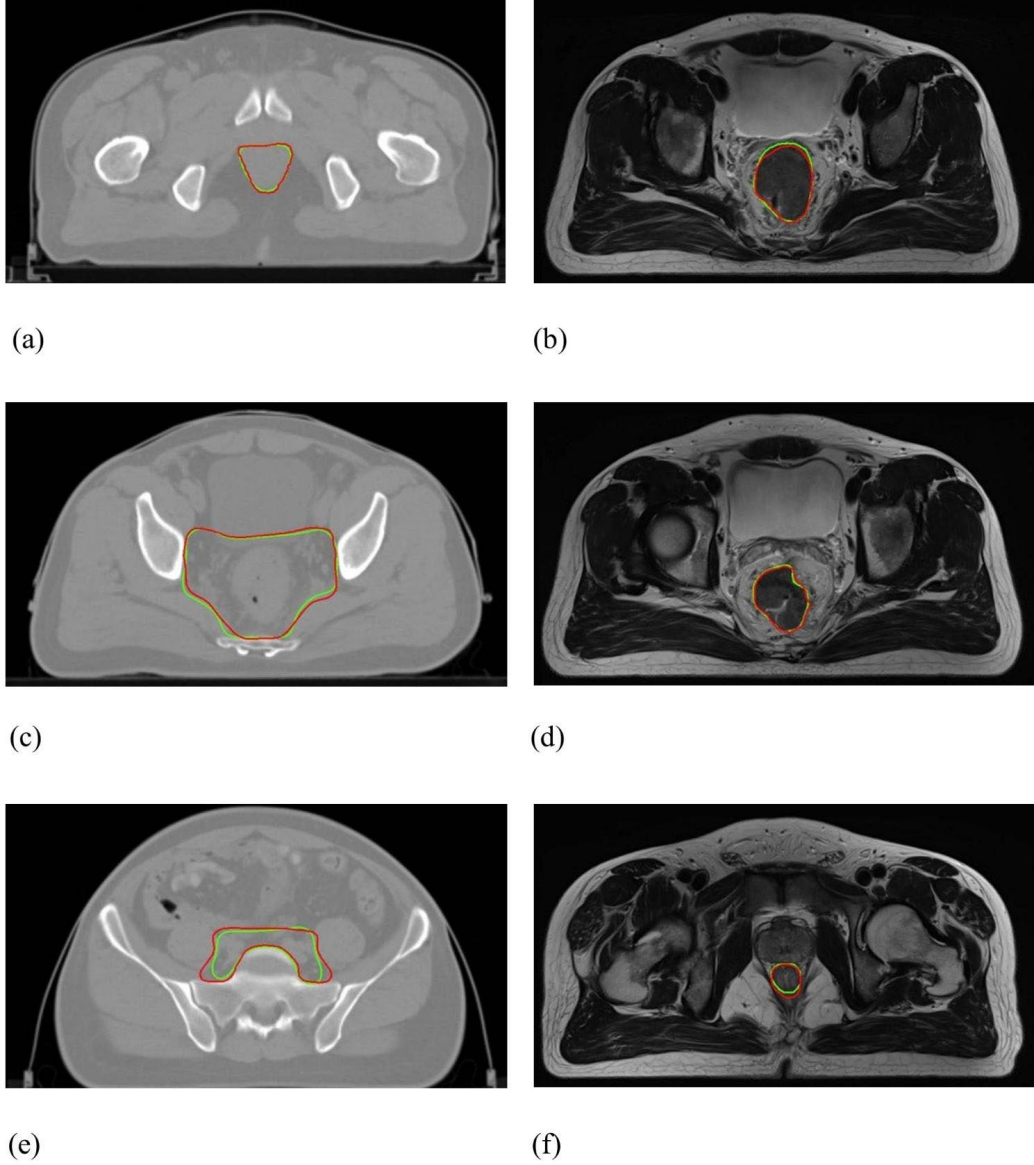
Representative axial illustration of Patient D: (**a**)&(**c**)&(**e**) CTV contours, where DSC = 0.88 and 95HD = 5.25 mm; (**b**)&(**d**)&(**f**) GTV contours, where DSC = 0.90 and 95HD = 5.22 mm. (GT-red line vs. DL-green line)

**Fig. 5:**
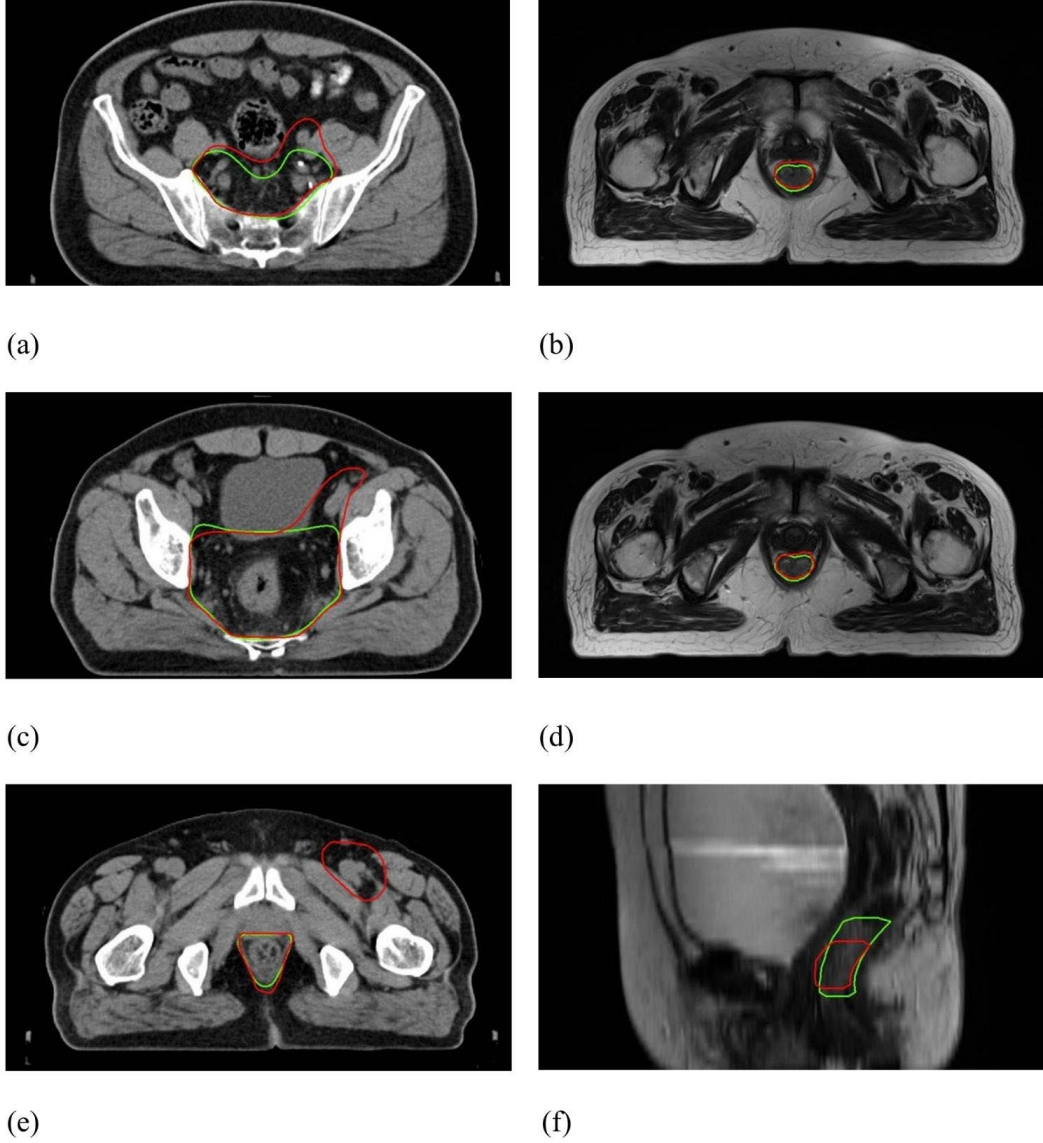
Representative illustration of outlier patient cases: (**a**)&(**c**)&(**e**) CTV contours of Patient K, where DSC = 0.84 and 95HD = 32.71 mm; (**b**)&(**d**)&(**f**) GTV contours of Patient S, where DSC = 0.64 and 95HD = 3.82 mm. (GT-red line vs. DL-green line)

Figure 3 shows the DSC and 95HD value distribution over the testing patient cohort, and Fig. 4 shows the CTV and GTV contours of a representative patient case (Patient D). For DL-based CTV segmentation (green squares in Fig. 3&), the DSC values range from 0.69 to 0.97 with mean ± SD as 0.85 ± 0.06, and the 95HD values range from 1.37 to 32.71 with mean ± SD as 7.75 ± 6.42. Two outlier data points in Fig. 3(b) are easily identified, i.e., Patient K with 95HD = 32.71 mm, and Patient B with 95HD = 15.50 mm. Representative axial images of Patient K are shown in Fig. 5(a)&(c)&(e), where the CTV-GT contour indicates this is a special case. The medical record shows that this patient was with perirectal lymph node invasion (LNI), and therefore mesorectal, sacral, internal iliac regions should be covered. This special treatment inevitable induced relatively large distance errors. This goes similarly with Patient B that required additional coverage of lymph nodes. When these two outliers are removed, the 95HD values range from 1.37 to 8.1 (5.93 ± 1.55).

For DL-based GTV segmentation (blue squares in Fig. 3), the DSC values range from 0.64 to 0.94 with mean ± SD as 0.87 ± 0.07, and the 95HD values range from 2.38 to 8.70 with mean ± SD as 4.07 ± 1.67. An outlier data point in Fig. 3(a) is easily identified, i.e., Patient S with DSC = 0.64. Representative GTV contours of Patient S are shown in Fig. 5(b)&(d)&(f). The GT and DL contours are highly similar in the middle axial planes, whereas DL contours exhibit over-coverage of surrounding tissue in the superior-inferior direction, which we did not figured out why yet and will be investigated in future.

### Level 2: expert scoring

**Fig. 6:**
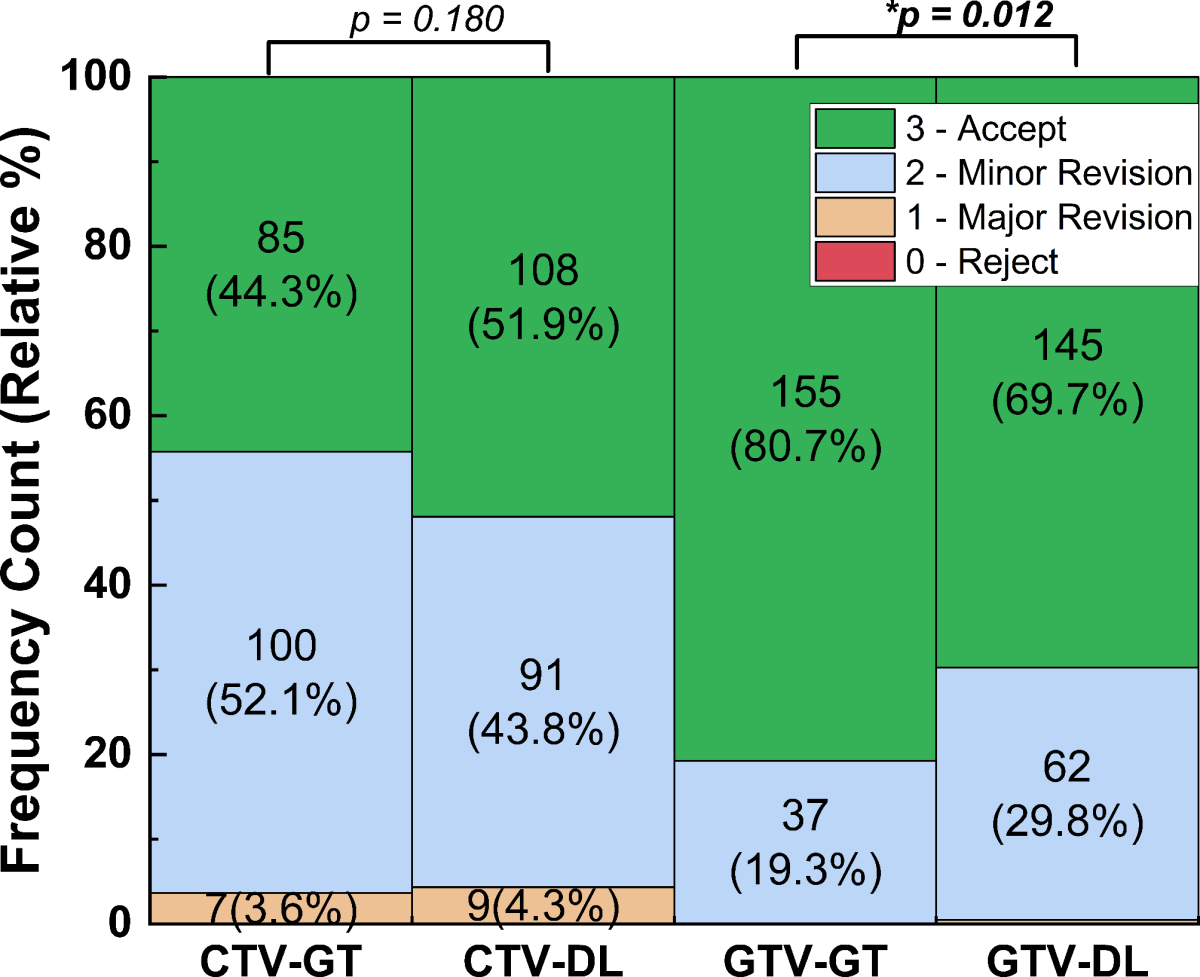
Frequency counts and relative (%) distribution of each grade in expert scoring. The p-values of GT vs. DL were calculated using Mann-Whitney U-test (* significance level < 0.05)

Figure 6 shows the distribution of expert scores on the blind and randomized CTV and GTV contours. Over the testing CTV contours, the cases accepted with no revision (Score = 3) account for 44.3% in GT and 51.9% in DL, and those requiring minor revision (Score = 2) account for 52.1% in GT and 43.8% in DL. The cases deemed as clinically viable (Score ≥ 2) are 96.4% in GT and 95.7% in DL. Besides, the cases requiring major revision (Score = 1) account for 3.6% in GT and 4.3% in DL, and none of GT and DL contours was rejected (Score = 0). The *p-value* between GT and DL scores is 0.180, indicating no significant difference.

Over the testing GTV contours, the cases accepted with no revision account for 80.7% in GT and 69.7% in DL, and those requiring minor revision account for 19.3% in GT and 29.8% in DL. In other words, 100% GT contours and 99.5% DL contours are deemed as clinically viable (Score ≥ 2). Besides, only one GTV-DL case requires major revision. The *p-value* between GT and DL scores is 0.012, indicating statistically significant difference.

### Level 3: head-to-head turing test

**Fig. 7:**
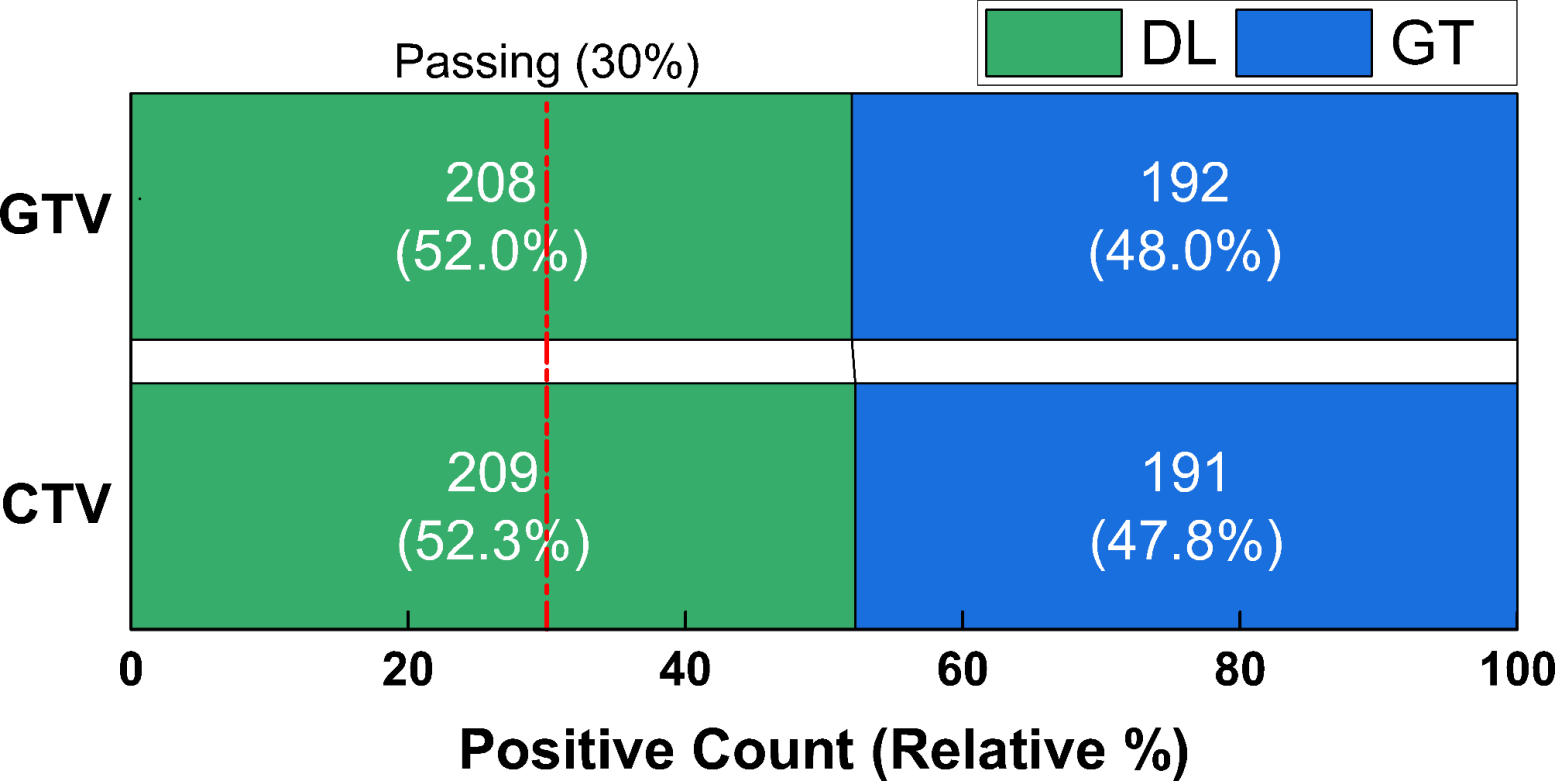
Head-to-head Turing test results of CTV and GTV (DL vs. GT). The passing threshold for DL contours is 30%

Figure 7 shows the result of the head-to-head Turing test, which is to reflect subjective preference between GT and DL contours. The proposed DL model passed the head-to-head Turing test (≥ 30%) for both CTV and GTV segmentation. Moreover, it is worth noting that DL contours prevail over GT contours in both CTV (DL vs. GT = 52.0% vs. 48.0%) and GTV (DL vs. GT = 52.3% vs. 47.8%) segmentation.

## Discussion

Accurate target delineation is crucial for ensuring optimal radiotherapy outcomes in patients with locally advanced mid-low rectal cancer. In this study, we developed a DL-based workflow for fully automated CTV and GTV delineation for rectal cancer neoadjuvant radiotherapy. The workflow used a divide-and-conquer strategy to address CTV segmentation in the CT domain and GTV segmentation in the MRI domain. We used a DpnUnet network as the backend for the DL models. The key advantage and significance for clinical work is, while there are a lot of DL-based studies focusing on OAR segmentation, our work focused on CTV and GTV segmentation for rectal cancer. This approach allowed oncologists to efficiently delineate both CTV and GTV volumes, representing a step forward towards DL-based fully automated target delineation. To validate the workflow, we designed a comprehensive three-level evaluation scheme that was multicenter-involved, blind, and randomized.

In Level-1, DSC and 95HD values over the testing cohort were calculated. For CTV segmentation, the DSC and 95HD values (mean ± standard deviation) were 0.85 ± 0.06 and 7.75 ± 6.42 mm. Compared with the previous studies by Wu et al. [14] (DSC = 0.90 ± 0.02, 95HD = 8.11 ± 1.93 mm) and by Men et al. [12] (DSC = 0.87), the performance of the proposed DL-based method seems inferior. Notably, there were seven cases with LNI in the training cohort, and two cases with LNI in the testing cohort. We assume that the cases with LNI were too limited for the model to gain pertinent capability, which can also explain the large distance errors in patient B&K. For GTV segmentation, the DSC and 95HD values were 0.87 ± 0.07 and 4.07 ± 1.67 mm. Compared with the study by Wang et al. [16] (DSC = 0.74 ± 0.14, HD = 20.44 ± 13.35 mm), the performance of the proposed method is essentially superior. The overall results demonstrate that the proposed DL-based workflow achieved promising performance in both CTV and GTV segmentation for rectal cancer neoadjuvant radiotherapy.

In Level-2, multicenter-involved, blind and randomized expert scoring was performed. The proposed DL-based method achieved 96.4% and 100% of clinically viable scores (≥ 2) for CTV and GTV segmentation respectively, indicating promising clinical applicability. While the DL contours showed a significant difference from the GT for GTV segmentation, since 99.5% of the DL contours were clinically viable, the clinical implication of being statistically different is negligible.

For the head-to-head Turing test in Level-3 evaluation, the proposed DL-based method not only passed the positive rate threshold (30%), but also was comparable with GT in expert subjective preference for both CTV and GTV segmentation. This excellent performance of the DL-based method indicates substantial potential in clinical application.

The evaluation results indicate two limitations of our work. First, we did give special arrangement of cases with LNI for model training and tuning, and consequently the current CTV model exhibits inadequate coverage of surrounding lymph nodes for patients with LNI. We will address this issue in future work to improve the overall capability of the proposed approach. Second, the current GTV model delivered one outlier case with major errors in superior and interior boundaries, indicating uncertainty in GTV boundary definition. The limitations implicate, if the current models were deployed for clinical application, clinicians should be careful of patient enrollment and manual review should be required in case of inadequate or excessive target coverage. In addition, only 20 patient cases were used for model performance testing. Future efforts will be made to enroll more patients to further verify the model robustness.

## Conclusion

The proposed DL-based workflow demonstrates promising accuracy and excellent clinical viability towards automated CTV and GTV delineation for rectal cancer neoadjuvant radiotherapy.

## Data Availability

The data that support this study are not openly available due to ethical and privacy concerns but are available from the corresponding author upon reasonable request.

## Abbreviations

95HD: 95th percentile Hausdorff distance
CNN: convolutional neural network
CT: computed tomography
CTV: clinical target volume
DDCNN: deep dilated CNN
DL: deep-learning
DPN: dual-path network
DSC: Dice similarity coefficient
GT: ground truth
GTV: gross tumor volume
MRI: magnetic resonance imaging
OAR: organ-at-risk
ROI: region of interest
RT: radiotherapy
SD: standard deviation

## References

[1] Chen W, Zheng R, Baade PD (2016). Cancer statistics in China, 2015. CA Cancer J Clin.

[2] Sung H, Ferlay J, Siegel RL (2021). Global Cancer Statistics 2020: GLOBOCAN estimates of incidence and Mortality Worldwide for 36 cancers in 185 countries. CA Cancer J Clin.

[3] Yang Y, Wang HY, Chen YK (2020). Current status of surgical treatment of rectal cancer in China. Chin Med J (Engl).

[4] Bonadeo FA, Vaccaro CA, Benati ML (2001). Rectal cancer: local recurrence after surgery without radiotherapy. Dis Colon Rectum.

[5] van Gijn W, Marijnen CA, Nagtegaal ID (2011). Preoperative radiotherapy combined with total mesorectal excision for resectable rectal cancer: 12-year follow-up of the multicentre, randomized controlled TME trial. Lancet Oncol.

[6] Yang J, Veeraraghavan H, Armato SG (2018). Autosegmentation for thoracic radiation treatment planning: a grand challenge at AAPM 2017. Med Phys.

[7] Glimelius B, Tiret E, Cervantes A, Arnold D, ESMO Guidelines Working Group (2013). Rectal cancer: ESMO Clinical Practice Guidelines for diagnosis, treatment and follow-up. Ann Oncol.

[8] Benson AB, Venook AP, Al-Hawary MM (2022). Rectal Cancer, Version 2.2022, NCCN Clinical Practice Guidelines in Oncology. J Natl Compr Canc Netw.

[9] Myerson RJ, Garofalo MC, Naqa E (2009). Elective clinical target volumes for conformal therapy in anorectal cancer: a radiation therapy oncology group consensus panel contouring atlas. Int J Radiat Oncol Biol Phys.

[10] Valentini V, Gambacorta MA, Barbaroet B (2016). International consensus guidelines on clinical target volume delineation in rectal cancer. Radiother Oncol.

[11] Men K, Dai J, Li Y (2017). Automatic segmentation of the clinical target volume and organs at risk in the planning CT for rectal cancer using deep dilated convolutional neural networks. Med Phys.

[12] Men K, Boimel P, Janopaul-Naylor J (2018). Cascaded atrous convolution and spatial pyramid pooling for more accurate tumor target segmentation for rectal cancer radiotherapy. Phys Med Biol.

[13] Larsson R, Xiong JF, Song Y et al. Automatic delineation of the clinical target volume in rectal Cancer for Radiation Therapy using three-dimensional fully convolutional neural networks. Annu Int Conf IEEE Eng Med Biol Soc. 2018:5898–901. 10.1109/EMBC.2018.8513506 30441678

[14] Wu Y, Kang K, Han C (2022). A blind randomized validated convolutional neural network for auto-segmentation of clinical target volume in rectal cancer patients receiving neoadjuvant radiotherapy. Cancer Med.

[15] Song Y, Hu J, Wu Q (2020). Automatic delineation of the clinical target volume and organs at risk by deep learning for rectal cancer postoperative radiotherapy. Radiother Oncol.

[16] Wang J, Lu J, Qin G (2018). Technical note: a deep learning-based autosegmentation of rectal tumors in MR images. Med Phys.

[17] Jin L, Chen Q, Shi A (2022). Deep learning for automated contouring of gross tumor volumes in Esophageal Cancer. Front Oncol.

[18] Yue Y, Li N, Shahid H (2022). Gross tumor volume definition and comparative Assessment for esophageal squamous cell carcinoma from 3D 18F-FDG PET/CT by Deep Learning-Based method. Front Oncol.

[19] Liu Z, Liu X, Guan H (2020). Development and validation of a deep learning algorithm for auto-delineation of clinical target volume and organs at risk in cervical cancer radiotherapy. Radiother Oncol.

[20] Xu L, Hu J, Song Y (2021). Clinical target volume segmentation for stomach cancer by stochastic width deep neural network. Med Phys.

[21] Müller D, Soto-Rey I, Kramer F (2022). Towards a guideline for evaluation metrics in medical image segmentation. BMC Res Notes.

[22] Liu Z, Chen W, Guan H (2021). An adversarial deep-learning-based model for Cervical Cancer CTV Segmentation with Multicenter Blinded Randomized Controlled Validation. Front Oncol.

